# A salivary chitinase of *Varroa destructor* influences host immunity and mite’s survival

**DOI:** 10.1371/journal.ppat.1009075

**Published:** 2020-12-04

**Authors:** Andrea Becchimanzi, Rosarita Tatè, Ewan M. Campbell, Silvia Gigliotti, Alan S. Bowman, Francesco Pennacchio

**Affiliations:** 1 Laboratorio di Entomologia “E. Tremblay”, Dipartimento di Agraria, University of Napoli “Federico II”, Portici (NA), Italy; 2 Istituto di Genetica e Biofisica “Adriano Buzzati Traverso”, Consiglio Nazionale delle Ricerche, Napoli, Italy; 3 Institute of Biological and Environmental Sciences, School of Biological Sciences, University of Aberdeen, Aberdeen, United Kingdom; 4 Istituto di Bioscienze e Biorisorse, Consiglio Nazionale delle Ricerche, Napoli, Italy; 5 Interuniversity Center for Studies on Bioinspired Agro-Environmental Technology (BAT Center), University of Napoli “Federico II”, Portici (NA), Italy; University of British Columbia, CANADA

## Abstract

*Varroa destructor* is an ectoparasite of honey bees and an active disease vector, which represents one of the most severe threats for the beekeeping industry. This parasitic mite feeds on the host’s body fluids through a wound in the cuticle, which allows food uptake by the mother mite and its progeny, offering a potential route of entrance for infecting microorganisms. Mite feeding is associated with saliva injection, whose role is still largely unknown. Here we try to fill this gap by identifying putative host regulation factors present in the saliva of *V*. *destructor* and performing a functional analysis for one of them, a chitinase (Vd-CHIsal) phylogenetically related to chitinases present in parasitic and predatory arthropods, which shows a specific and very high level of expression in the mite’s salivary glands. Vd-CHIsal is essential for effective mite feeding and survival, since it is apparently involved both in maintaining the feeding wound open and in preventing host infection by opportunistic pathogens. Our results show the important role in the modulation of mite-honey bee interactions exerted by a host regulation factor shared by different evolutionary lineages of parasitic arthropods. We predict that the functional characterization of *Varroa* sialome will provide new background knowledge on parasitism evolution in arthropods and the opportunity to develop new bioinspired strategies for mite control based on the disruption of their complex interactions with a living food source.

## Author summary

*Varroa destructor* is a parasitic mite of honey bees and a major driver of honey bee colony losses. The feeding mites inject a salivary blend of poorly known molecules, which regulate host physiology. Here, we have identified *in silico* putative host regulation factors occurring in *Varroa* saliva and characterized the functional role of a highly expressed chitinase, which is conserved across different evolutionary lineages of parasitic arthropods. This enzyme influences host immune response and mite’s survival. An in-depth functional analysis of *Varroa* saliva will shed light on parasitism evolution in arthropods and will pave the way towards the development of new bioinspired strategies for mite control.

## Introduction

Honey bee (*Apis mellifera*) colony losses and their negative consequences, both at ecological and economic level, have been widely reported in different regions of the world [1]. Several monitoring programs indicated that high loads of parasites and pathogens largely contribute to this problem [2]. In particular, the association between *Varroa destructor* and Deformed Wing Virus (DWV) accounts for a large majority of honey bee colony losses [3].

The mite *V*. *destructor* is an obligate ectoparasite of honey bees, which feeds on body fluids through a wound made on the integument of the host [4,5]. This parasite has a severe impact on host physiology [6], causing reduction of weight at emergence [7], as well as shortening of life span [8], and acts as a vector of several viral pathogens, which establish very tight associations with *V*. *destructor* [9–11].

During the mite reproductive phase, inside capped cells, adult females spend nearly an hour to produce a wound on the integument of honey bee pupae, usually localized on the second abdominal segment, through which they can feed, along with their progeny, over a relatively long time interval [12]. The honey bee cuticle is perforated by mite’s chelicerae [13], and the hypostome is used to inject saliva and to suck host body fluids [14,15]. This ectophagous feeding habit has to mitigate or evade the immune reaction of the host in order to allow effective food uptake and use [16]. Moreover, mite feeding through an open wound exposes the host to the risk of infection by opportunistic pathogens [17]. The mite strategy to successfully overcome these problems has received limited attention so far, but the available evidence in a number of host-parasite associations among arthropods indicates that the saliva can play an important role [18].

Little is known about *Varroa* saliva injected into the host by feeding mites, but available experimental evidence suggests that it is very likely involved in host regulation and exploitation. One of the first functional studies on *V*. *destructor* saliva [19] reported that mite’s saliva is able to disrupt cellular immune response by haemocytes of the caterpillar *Lacanobia oleracea*. More recently, it was reported that degradation of fat body cells beneath the pierced intersegmental membrane of parasitized adult bees is induced by saliva injection [20]. Another recent study identified in the saliva of *V*. *destructor* a protein component that is toxic for pre-imaginal stages of *Apis cerana* and promotes an increase of DWV titer in *A*. *mellifera* [21]. Finally, the proteomic analysis of the mite saliva, collected with an *in vitro* feeding system, identified proteins putatively acting as virulence factors, antimicrobials or exerting antioxidant and detoxification functions [22].

Surprisingly, so far, very little research efforts have been devoted to the functional characterization of saliva components released by *Varroa* mites feeding on honey bees. These studies can generate new knowledge on the molecular mechanisms regulating the interactions in this important host-parasite association and will pave the way towards the development of promising RNAi-based control strategies for *Varroa* mite [23,24].

Here we try to fill this research gap, using literature mining and bioinformatics tools to identify putatively secreted proteins of *V*. *destructor* that share significant sequence similarity with host regulation factors reported in the saliva of Acarina and venoms of Hymenoptera, and assessed their effective expression in salivary glands by *in situ* hybridization. This selective approach allowed the identification of a putative virulence factor, specifically and highly expressed in the salivary glands of *V*. *destructor*, which was subsequently studied from a functional point of view, by analyzing both its impact on mite survival and its host regulation effects on honey bees.

## Results

### Putative host-regulation factors in the salivary glands of *Varroa destructor*

**Fig 1:**
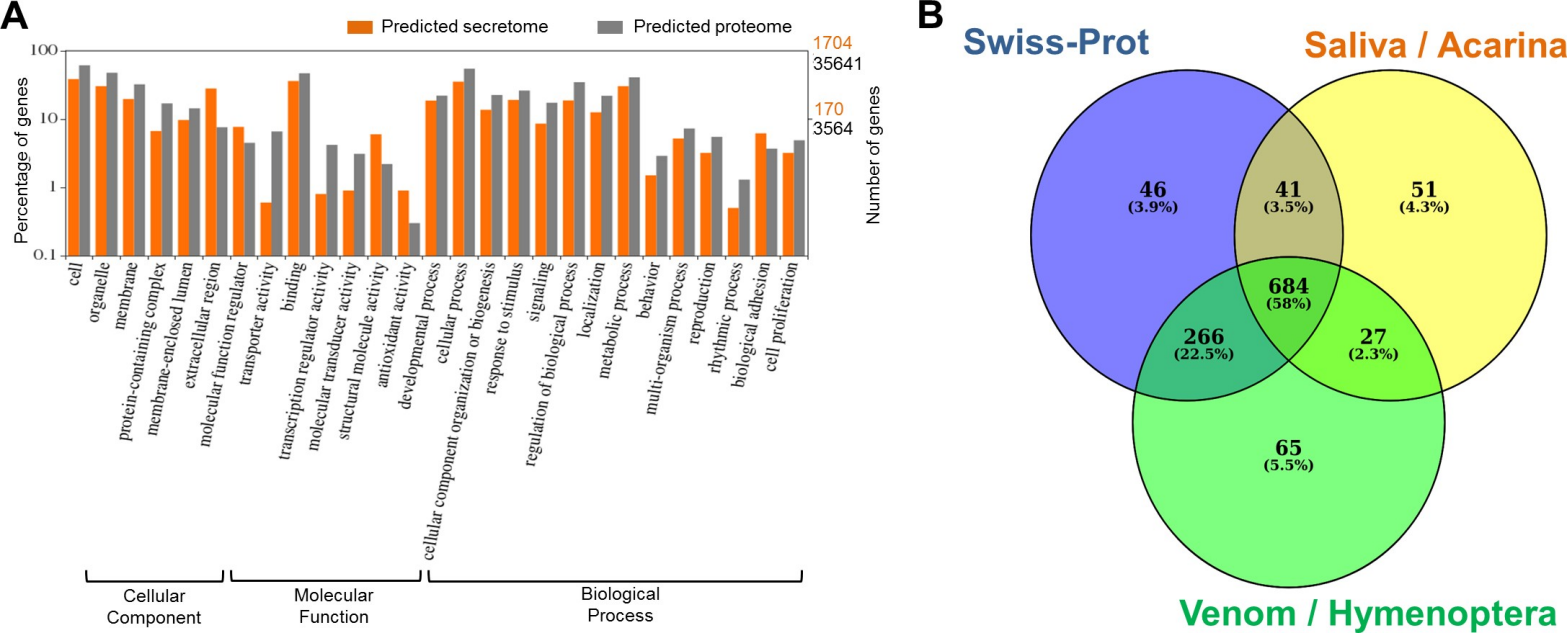
Annotation of the predicted *Varroa destructor* secretome. (A) Abundance of Gene Ontology terms Level 2 from the predicted secretome (orange) and the whole predicted protein set (gray) of *V*. *destructor* plotted using WEGO v. 2.0. Percentage and number of genes in Log_10_ scale are reported on left and right Y-axes, respectively. Of the total 1,704 proteins in the predicted secretome, only 1,030 matched in the Swiss-Prot database and led to the GO terms represented above (orange). WEGO analysis showed that “extracellular region”, “molecular function regulator”, “structural molecule activity”, “antioxidant activity” and “biological adhesion” are overrepresented GO terms in the predicted secretome, compared to the whole predicted proteome. (B) Venn diagram showing number and percentage of secretome sequences matching in three different databases. Swiss-Prot (blue), salivary proteins of Acarina (yellow) and venom proteins of Hymenoptera (green). Venom proteins of Hymenoptera species were downloaded from NCBI using keyword ‘venom AND Hymenoptera [organism]’), while “saliva-related” tick’s and mite’s proteins were obtained using keyword ‘saliva AND Acarina [organism]’. The predicted secretome was blasted using an E value cut-off of 10^−5^. Venn diagram of annotated proteins was plotted using the online tool Venny v. 2.1.

Given the convergent origin of toxins across the entire metazoan spectrum, we used an *in silico* approach to predict the mite secretome and to infer putative homologies with proteins from salivary blends of Acarina (ticks and mites) and venoms of Hymenoptera (including parasitic wasps). The 35,641 proteins annotated in the *V*. *destructor* genome included 1,704 sequences with a signal peptide and lacking transmembrane domains (i.e. putatively secreted) (S1 File). Gene ontology enrichment analysis performed on this set of sequences is reported in Fig 1A. BLASTp searches using the predicted secretome as the query resulted in a subset of 1,180 proteins having a match to at least one of the three searched databases (Swiss-Prot, saliva of Acarina and venom of Hymenoptera) as shown in Fig 1B.

The resulting high number in the mite’s secretome of homologues of proteins present in venom and salivary blends of parasitic arthropods, often obtained through recruitment and slight modification of genes involved in key-regulatory processes, indicates the occurrence of similar evolutionary patterns also in *Varroa*.

In order to prioritise a set of putative salivary effectors to be functionally studied, we mined literature relative to the well-studied salivary blends of ticks and selected three *V*. *destructor* secreted proteins having a match in both the saliva of Acarina and venom of Hymenoptera: α-Macroglobulin [25], Aspartic Protease [25] and Chitinase [26] (S1 Table). Notably, these proteins were found in an in-house proteomic atlas of *V*. *destructor* saliva, available at the University of Aberdeen.

**Fig 2:**
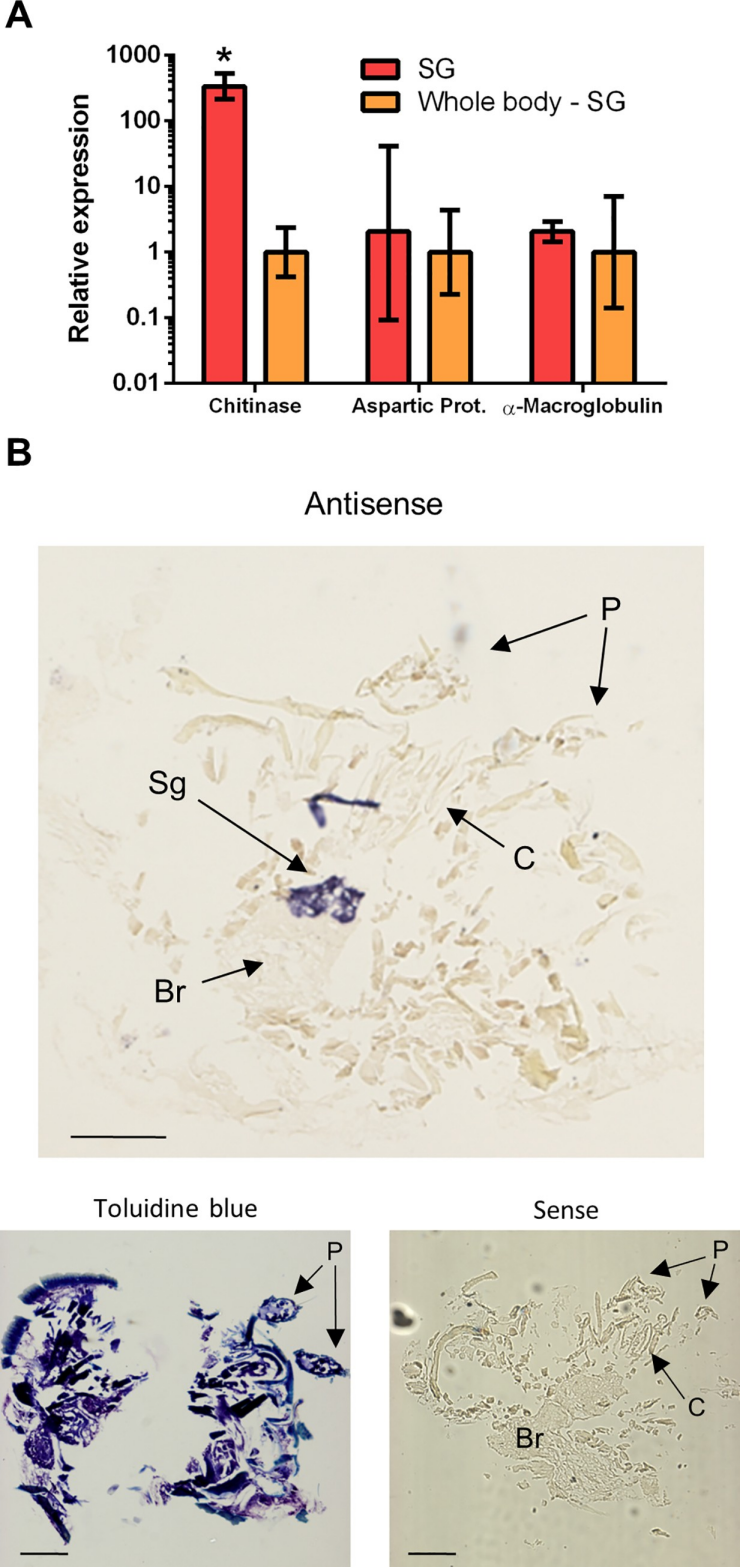
Salivary gland expression of putative host regulation factors found in the predicted secretome of *Varroa destructor*. (A) Relative expression data of 3 selected candidates are presented as mean fold changes of 3–4 independent biological replicates. Each replicate consisted of a pool of 5–10 mites and comprised two samples: salivary glands (SG), and rest of the whole body, deprived of salivary glands (Whole Body–SG). Values on Y-axis are reported in Log_10_ scale. Error bars represent standard deviation (SD). Statistically significant differences are denoted with an asterisk (P < 0.005). (B) *In-situ* hybridization of DIG-labeled RNA probe for the salivary chitinase (*Vd-CHIsal*). Salivary glands hybridized with the antisense probe showed positive blue signals, while neither background nor unspecific signals were observed when the sense probe was used (negative control). B: Brain; C: Chelicera; P: Pedipalps; Sg: Salivary glands. Scale bars: 100 μm.

The expression profile of these putative host regulation factors, obtained by qRT-PCR, demonstrated a ~340-fold enrichment of the chitinase transcript (*Vd-CHIsal*) in salivary glands of adult *Varroa* females, as compared with the rest of the body (Student’s t-test: P < 0.005) (Fig 2A). The other candidates also demonstrated a trend of higher expression in the salivary glands (between 2- and 5-fold), but the recorded differences were not statistically significant. Tissue-specific expression of *Vd-CHIsal* was further corroborated by *in situ* hybridization experiments on sectioned *Varroa* mites, showing intense and specific signals that were completely restricted to the salivary glands (Fig 2B). Therefore, Vd-CHIsal was selected for a phylogenetic and functional characterization.

### Sequence analysis and phylogenetic reconstruction of Vd-CHIsal

Vd-CHIsal is a protein of 389 amino acids with a predicted molecular weight of 43 kDa. A protein–protein BLAST search using the amino acid sequence of Vd-CHIsal (endochitinase-like, GenBank: XP_022673141.1) demonstrated similarities with a number of chitinases from arthropod species classified in family 18 of the glycoside hydrolases, such as those of *Varroa jacobsoni* (GenBank: XP_022691493.1, 100% query cover, 99% identity), *Tropilaelaps mercedesae* (GenBank: OQR72877.1, 98% query cover, 71% identity) and *Galendromus occidentalis* (GenBank: XP_003747412.1, 99% query cover, 59% identity). Notably, while sharing with the other chitinases the presence of the glycoside hydrolase family 18 catalytic domain, Vd-CHIsal misses the chitin-binding Peritrophin-A domain, which is also absent in the chitinase found in the venom of the parasitic wasp *Chelonus inanitus* (GenBank: CBM69270.1) (S1 Fig).

**Fig 3:**
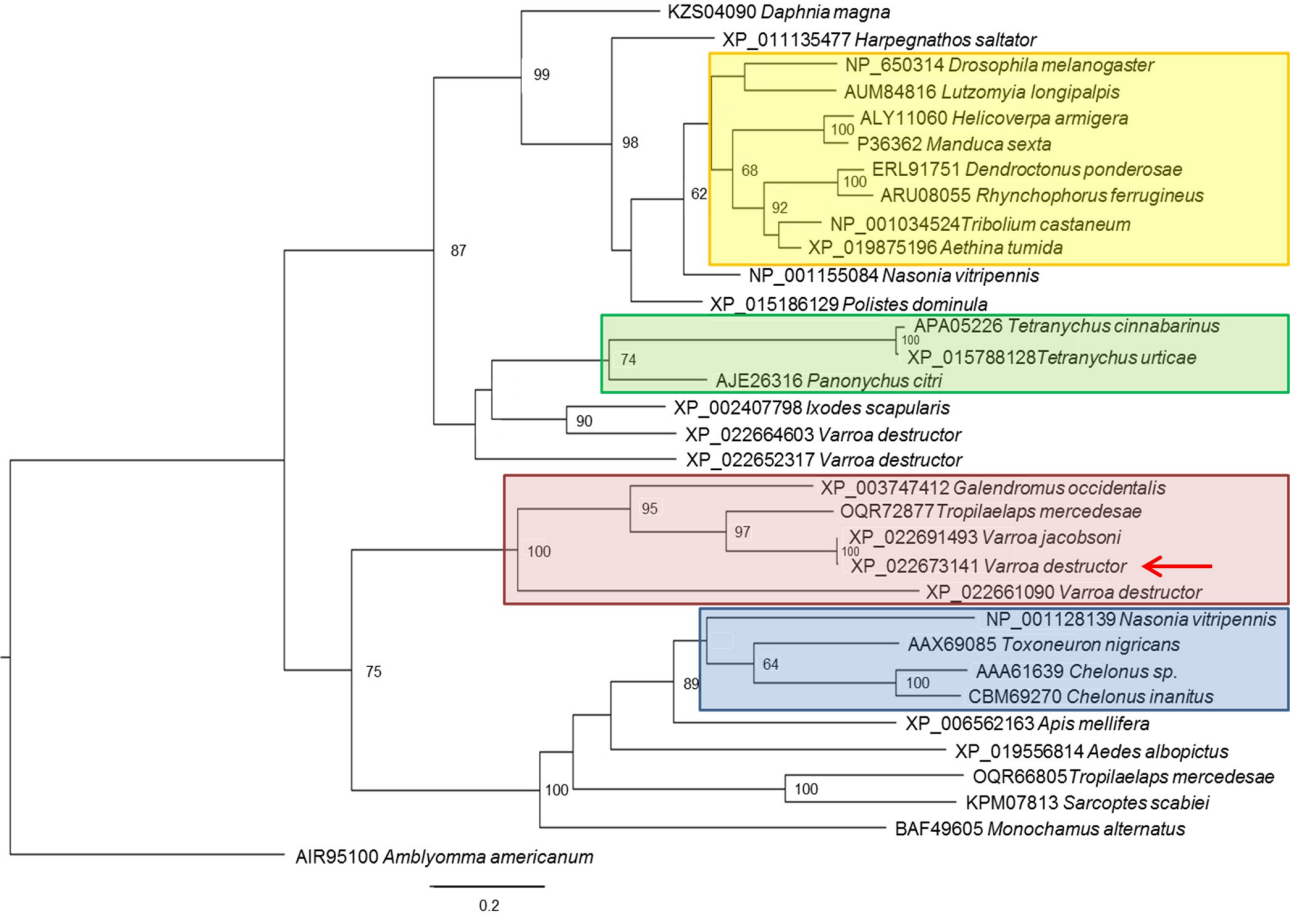
Maximum-likelihood phylogenetic tree of Vd-CHIsal protein. Bootstrap support values ≥60% are indicated. The accession number of each sequence is followed by the taxon name. Vd-CHIsal is indicated by a red arrow. The branch highlighted in pink represents the monophyletic group which Vd-CHIsal forms with few chitinases of predator and parasitic mites. Other highlighted branches represent chitinases with a known role in parasitoid-host interactions (blue), chitinases of phytophagous mites (green) and chitinases with a known function in the molting process (yellow). A chitinase of *Amblyomma americanum* (GenBank: AIR95100) showed the longest branch in the unrooted tree and was used as the outgroup reference.

Phylogenetic analysis of 32 aligned chitinases, performed with maximum-likelihood, indicated, with bootstrap support, that Vd-CHIsal forms a monophyletic group with chitinases of predator and parasitic mites (Fig 3). In this clade, *V*. *destructor* chitinases are represented only by Vd-CHIsal and another sequence (GenBank: XP_022661090), while the other putative homologs of Vd-CHIsal identified in the same species are clustered in different groups. It is interesting to note that Vd-CHIsal resulted more closely related to parasitoid chitinases, reported as host regulation factors present in venom or released by teratocytes, than to chitinases of phytophagous mites and chitinases with a known function in the molting process of arthropods (Fig 3).

### Impact of *Vd-CHIsal* silencing on mites

**Fig 4:**
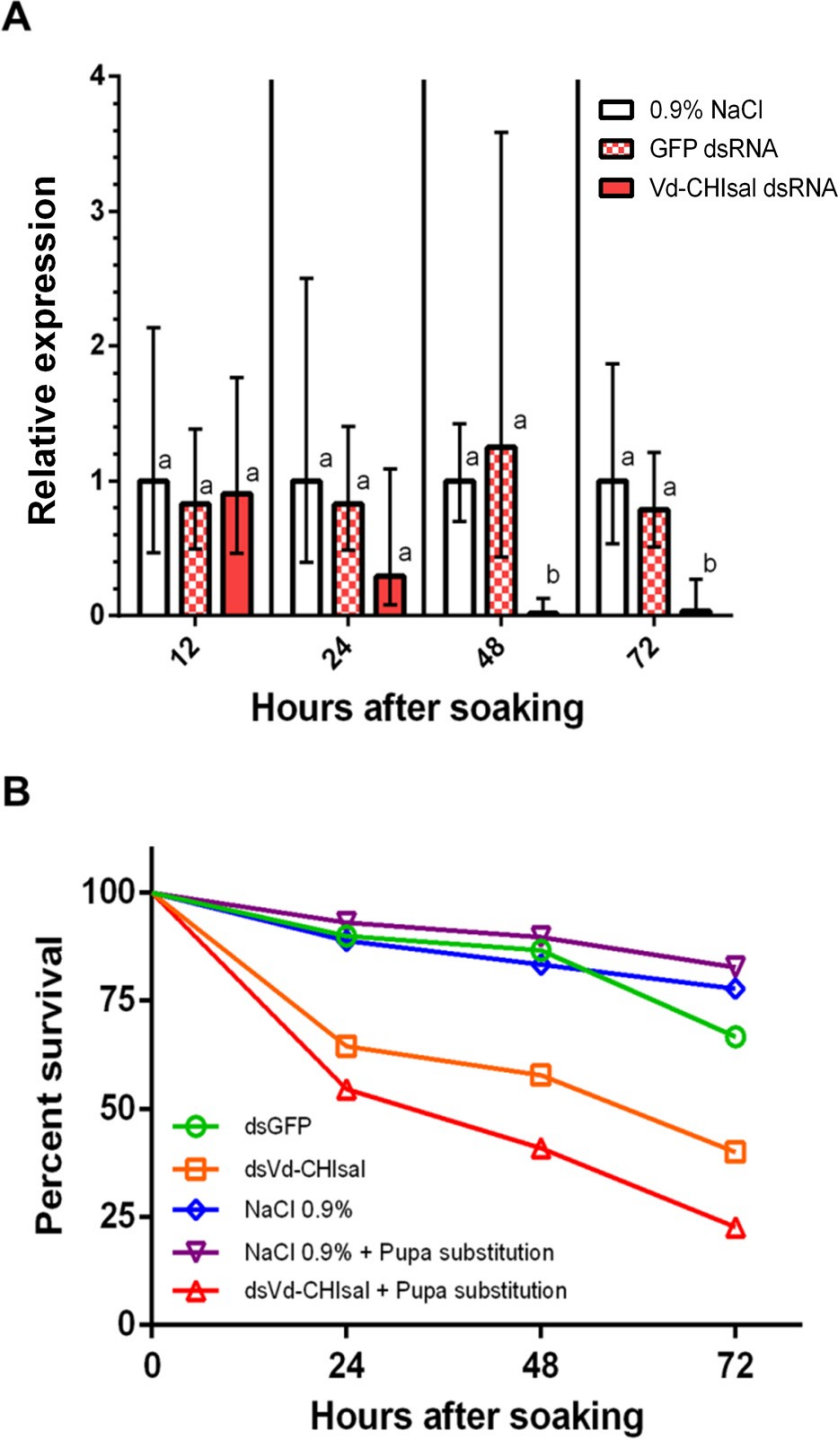
Survival of *Varroa destructor* as affected by RNAi-mediated silencing of the gene encoding Vd-CHIsal. (A) Relative expression of *Vd-CHIsal* after mite soaking in a dsRNA solution. qRT-PCR data are presented as mean fold changes of 7 independent biological replicates. Each replicate consisted in a pool of 2 mites. Each time point was separately analyzed and the 0.9% NaCl control sample was used as calibrator. Mean dCt values within each time point were compared by one-way ANOVA followed by Tukey’s post-hoc test. Mean values denoted with different letters are significantly different. Error bars represent standard deviation (SD). (B) Kaplan-Meier survival curves of mites soaked in a solution of dsRNA targeting *Vd-CHIsal*. Saline controls (0.9% NaCl), GFP dsRNA and *Vd-CHIsal* dsRNA soaked mites were individually maintained on the same host pupa throughout the whole duration of the assay. Subjects at risk were 18, 30 and 45 for 0.9% NaCl, GFP dsRNA and *Vd-CHIsal* dsRNA treatments, respectively. In a concurrent set of trials, host pupae were replaced every 24 h for both saline controls (0.9% NaCl) and *Vd-CHIsal* dsRNA soaked mites. Subjects at risk were 29 and 22 for 0.9% NaCl and *Vd-CHIsal* dsRNA, respectively. Statistical details are in the text.

Mite soaking in a *Vd-CHIsal* dsRNA solution induced a statistically significant gene silencing 48 h after the treatment (98.3% reduction), which persisted at 72 h (96.6% reduction) (Fig 4A and S2 Table). No statistically significant difference in *Vd-CHIsal* expression was observed between mites treated with 0.9% NaCl and mites treated with *GFP* dsRNA, at any time point (S2 Table).

Mites soaked in saline solution supplemented with dsRNA targeting *Vd-CHIsal* had a significantly lower survival rate than mites soaked in either control *GFP* dsRNA (log rank test: *X*_2_ = 6.086; P = 0.0136) or control saline solution (log rank test: *X*_2_ = 6.611; P = 0.0101), which did not differ between them (Fig 4B). When the host pupa was replaced every 24 h the mortality of *Vd-CHIsal* dsRNA-soaked mites was even more pronounced compared to controls (log rank test: *X*_2_ = 18.21; P<0.0001) (Fig 4B). In order to check any possible confounding effect due to a different level of basal infection, the DWV titer of pupae used in these experiments was scored by qRT-PCR and resulted similar in all samples (S2 Fig).

### Impact of *Vd-CHIsal* silencing on infested honey bee pupal gene expression

To investigate the possible effects of Vd-CHIsal on honey bees, we studied the host transcriptional response upon artificial infestation with *Varroa* mites producing Vd-CHIsal deficient saliva (KD), compared to control mites, delivering saliva with the whole repertoire of salivary factors (WS). Non-parasitized control pupae (NP) were used as the reference sample. Because we were interested in the effects of salivary factors on early steps of host regulation, the samples were those collected 24 h after the onset of mite infestation in the survival experiments reported above and untreated non-parasitized controls.

**Fig 5:**
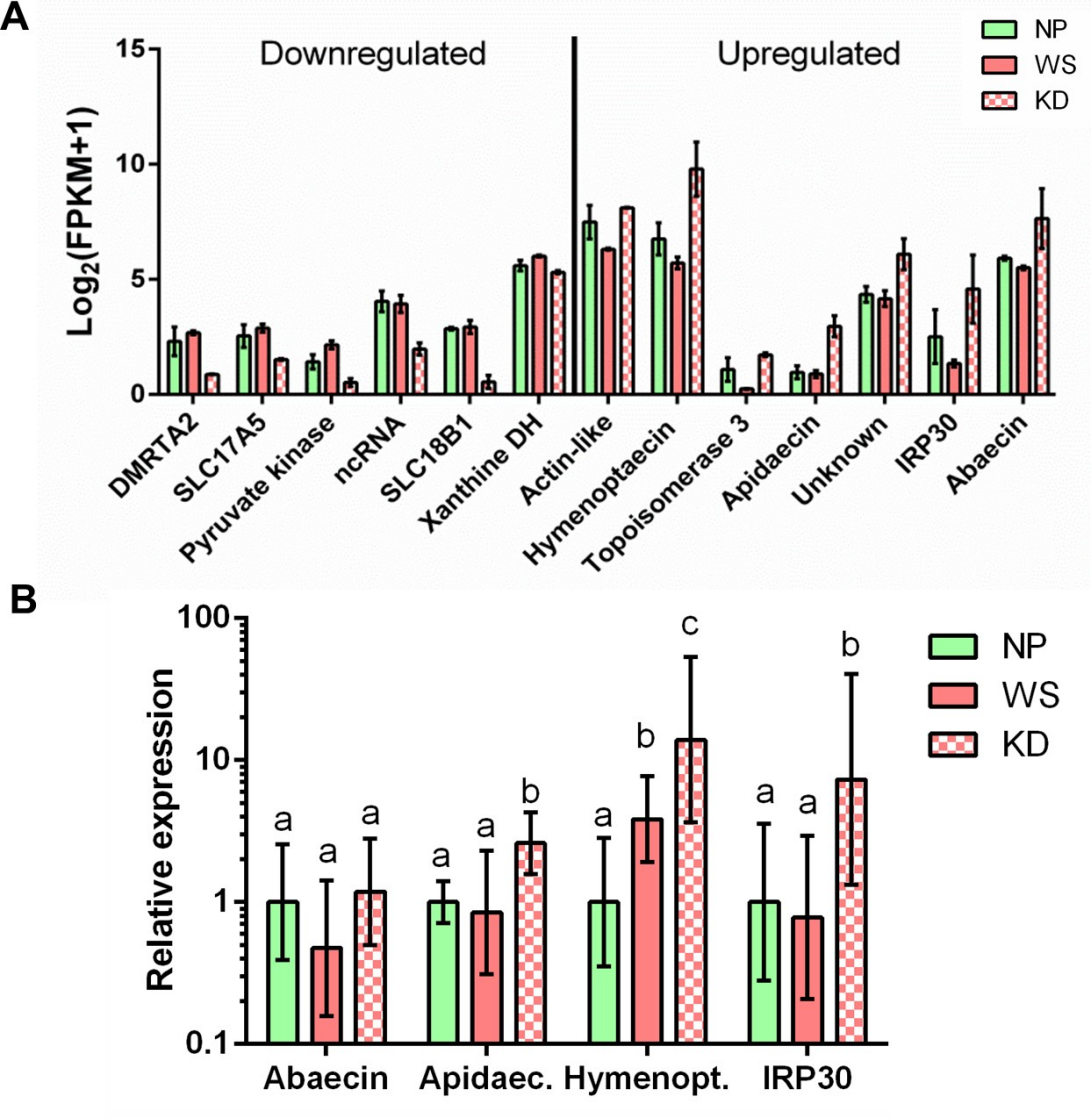
Differentially expressed genes in honey bee pupae artificially infested with mites delivering saliva with the full repertoire of proteins or lacking Vd-CHIsal. (A) Differential expression of 13 honey bee genes, as affected by presence of Vd-CHIsal in the saliva (KD/WS). DESeq2 adjusted *P* was < 0.05 and FDR was set at 5%. Log transformed mean FPKM values are reported on Y axis. For each experimental condition 3 separate pupae were analyzed. Error bars represent SD. Summary data sheets of differential expression analysis are presented in S2 and S3 Tables. (B) Relative expression of immune genes in honey bee pupae as affected by *Vd-CHIsal* expression in *Varroa destructor* infesting mites. Each mean value is obtained on 7–10 pupae, individually analyzed. Results of qRT-PCR are presented as mean fold changes relative to non-infested pupae used as calibrator. Values on Y axis are reported in Log_10_ scale. Error bars represent standard deviation (SD). Mean values were compared by one-way ANOVA, followed by Tukey post-hoc test, and values statistically different are denoted with different letters (P<0.05). Details of statistical analyses are presented in S4 Table. NP: non-parasitized controls; WS: pupae infested with mites soaked in saline solution; KD: pupae infested with mites soaked in a solution of *Vd-CHIsal* dsRNA.

*Vd-CHIsal* knockdown in mites’ salivary glands had a limited and targeted effect on gene expression of honey bee pupae. Indeed, only 13 genes showed significantly altered expression profiles in response to the injection of saliva with a strongly reduced content of Vd-CHIsal (KD) compared to controls (WS) (DESeq2 adjusted *P* < 0.05). The pairwise comparison between KD and WS led to the identification of 6 downregulated and 7 upregulated genes (Fig 5A, S3 and S4 Tables). The genes which were downregulated in KD encode for a pyruvate kinase, two MFS type transporters (SLC17A5 and SLC18B1), a xanthine dehydrogenase and a Doublesex-Mab-3 Related Transcription factor A2 (DMRTA2). Moreover, we observed the downregulation of a ncRNA (LOC107964741). The bee pupal genes that were upregulated upon infestation with *Vd-CHIsal* knockdown mites (i.e. when the Vd-CHIsal content in the saliva is expected to be greatly reduced) encode for molecules involved in the immune response against microbial pathogens (abaecin, apidaecin, hymenoptaecin and IRP30).

In order to experimentally corroborate this latter result, which appeared to be particularly relevant in the modulation of host-parasite interaction, immune genes found to be differentially expressed by RNAseq analysis were further studied by qRT-PCR on a separate larger number of biological replicates of WS, KD and NP pupae. A statistically significant upregulation was evident for KD in 3 out of the 4 examined genes, with only *abaecin* showing no differential expression in any experimental condition considered (Fig 5B and S5 Table). As above, this result was not affected by different viral loads (S2 Fig).

## Discussion

Parasites of living organisms have developed a wealth of strategies to deliver repertoires of molecules inside the host, in order to regulate its physiology, basically to circumvent the immune barriers and to allow its nutritional exploitation [27,28]. The study of natural weapons developed by foreign invaders and of the mechanisms underlying their mode of action in the host is a fascinating research area which can pave the way towards new strategies of parasite control based on learning from natural processes shaped by a long co-evolutionary history [29].

*Varroa* is one of the major threats of honey bees, an obligate ectoparasite which, during feeding activity on the host, injects salivary secretions only partly identified so far [19,21,22] and still poorly investigated from a functional point of view. The identification and characterization of these virulence factors and their coding genes will offer new insights into the molecular basis of *Varroa*-honey bee interactions, on which to develop new sustainable strategies of mite control. In particular, the development of RNAi-based control strategies, already suggested a few years ago [23], appears now even more promising after the recent discovery of effective symbiont-mediated RNAi [24].

Here, we have used an *in silico* approach to identify *V*. *destructor* candidate host regulation factors present among the salivary proteins. We selected a chitinase (Vd-CHIsal) that is highly and specifically expressed in the mite salivary glands, and analyzed its functional role in the regulation of the mite-honey bee interaction. By assessing the effect of the whole repertoire of salivary factors, lacking the investigated Vd-CHIsal, our analysis provided information of the impact exerted by this protein both on mite and honey bee, under *in vivo* conditions.

Based on the evolutionary hypothesis of convergent recruitment of proteins in venom and feeding secretions across multiple animal lineages [30], we hypothesized the possible presence in the *Varroa* secretome of proteins found in the well-studied ticks’ saliva and in the venom blends of parasitic wasps (Hymenoptera). Indeed, about 60% of BLAST annotated proteins in the predicted *V*. *destructor* secretome was shared with both salivary proteins of Acarina and venom proteins of Hymenoptera. In the case of Vd-CHIsal, which is specifically expressed in salivary glands, further BLAST searches in the NCBI database and phylogenetic reconstruction of the amino acid sequence identified a close relationship with chitinases found in the venoms of parasitoid wasps. These results are well in tune with the available evidence reporting that ticks and parasitoid wasps, as well as other venomous animals can secrete and inject into the host similar virulence factors to subdue them and/or redirect their physiology. Examples of this convergence are represented by phospholipases A_2_ [31,32], serine protease inhibitors [33] and chitinases [26,34]. Many parasitism factors are, in fact, proteins that participate in fundamental physiological processes, well conserved in the animal kingdom, which may represent a particular example of intergenome active elements with disruptive effects when introduced by a different organism, sometimes after a slight modification [30]. Moreover, our analysis reported 143 secreted proteins matching the databases of arthropods’ secretions, but showing no significant similarity with sequences in Swiss-Prot. We may likely have parasitism factors among these unannotated proteins, which are obviously more difficult to characterize from a functional point of view, though observation of phenotypes following gene knockdown may aid in elucidating the role and properties of these unknown proteins.

Among the many candidates, we selected three genes on the basis of literature reports on their possible involvement in the modulation of host-parasite interaction in ticks and identified their putative homologs in *V*. *destructor*. The expression profile of the three selected genes showed that only *Vd-CHIsal* is significantly overexpressed (340-fold increase) in the mite salivary glands, compared to the rest of the body, clearly indicating high tissue-specificity, a common trait shared by genes encoding the main bioactive components of salivary and venom blends [35]. Although the other two genes investigated, encoding α-Macroglobulin and Aspartic Protease, are expressed to a similar extent in other tissues in the experimental samples, we cannot exclude that the observed trend of transcript enrichment in salivary glands, even though statistically not significant in our analysis, can be functionally relevant, and indicates that these proteins can have a role in the host regulation and exploitation, which is probably worth of further research efforts.

Chitinases are glycosyl hydrolases mediating the degradation of chitin, which is a structural component of protective biological matrices, such as arthropod exoskeleton and fungal cell wall [36]. In arthropods, chitinases play a central role in cuticle degradation during molting and a defensive role against parasites, such as fungi and nematodes [37]. The presence of chitinase activity in adult *V*. *destructor* has already been described [38,39]. The high activity of chitinase in the *V*. *destructor* salivary glands was speculated to play a role in the hydrolysis of the host chitin and in keeping open the wound that serves for feeding the female-mother and her progeny and that is known to fail to seal during the feeding cycle [39].

To assess if Vd-CHIsal has a role in the mite feeding, we generated Vd-CHIsal deficient mites through dsRNA delivery and performed artificial infestations of honey bee pupae. Mite survival decreased by 60% when Vd-CHIsal was knocked down. Notably, the increase in mortality was greater when the host was replaced every 24 hours and the mite could not use the same feeding wound but was forced to create a new feeding site daily. These pieces of experimental evidence strongly suggest that feeding is impaired upon *Vd-CHIsal* gene silencing. Piercing the honey bee pupa cuticle is time-consuming and energetically demanding [12]. Although the opening of the feeding site is due mainly to the mechanical rupture of cuticle by the mite’s chelicera [40], we propose that Vd-CHIsal is likely involved in this initial step of wound formation and can contribute to feeding site remaining pervious. Actually, this enzyme could be part of a repertoire of salivary enzymes which are able to interfere with the damage signaling pathways underlying the early steps of clotting and wound healing [41,42]. This hypothesis is also supported by a protein atlas of different developmental stages of *V*. *destructor*, which indicates the presence of *Vd-CHIsal* only in the adult females [43], which is the stage producing a long-lasting feeding site, used also by the offspring [12].

Vd-CHIsal exhibits the conserved sequence motifs of GH18 chitinases (DXXDXDXE), which includes the active site of the enzyme [44]. Vd-CHIsal lacks a putative chitin-binding domain, though this is not essential for the enzymatic activity in arthropods [45]. A similar domain loss is also observed for a chitinase found in the venom of the parasitoid wasp *Chelonus inanitus* [34]. Interestingly, two enzymatically inactive chitinases lacking chitin-binding domains were suggested to be involved in the maintenance of a feeding site and blood meal acquisition in the tick *Amblyomma americanum* [26], in a scenario where it is unclear how a chitinolytic saliva would be beneficial. A thorough biochemical characterization of Vd-CHIsal is necessary to elucidate the molecular mechanism underpinning its role in the establishment and maintenance of a Varroa mite feeding site on honey bee.

To assess the impact of *Vd-CHIsal* silencing on honey bees, we studied the transcriptional response in bee pupae following mite artificial infestation. RNAseq analysis showed that *Vd-CHIsal* silencing is associated with a lower transcription level of honey bee genes involved in metabolism and transport (pyruvate kinase, two transporters belonging to the Major Facilitator Superfamily, a xanthine dehydrogenase), while upregulated genes are largely involved in immunity. Hymenoptaecin and apidaecin are antimicrobial peptides under the Toll pathway [46] and their upregulation is associated with immune response to fungi and bacteria [47,48]. The leucine-rich repeat (LRR) protein termed IRP30 is released in honey bee’s hemolymph by the fat body in response to injection of different components of microbial cell walls, such as lipopolysaccharides (gram-negative bacteria) and laminarins (fungi), but its release is not elicited by aseptic wounds [49]. A gene with unknown function (LOC100578816) encodes a 100 AA long peptide, putatively secreted, which shows 34.2% identity with a disintegrin metalloproteinase (*Aspergillus nomius*) and 36.7% identity with a Multiple epidermal growth factor-like domains 10 (*Crassostrea gigas*). The expression of this gene was found to be upregulated upon viral infection [50], thus suggesting a possible role in immune response.

The transcriptional profile of these immune genes indicates the occurrence of a stronger immune challenge in pupae infested by *Vd-CHIsal*-silenced mites, likely mediated by infection of microorganisms, which in natural (i.e. non-silenced) mite infestations is in some way moderated by Vd-CHIsal. Alternatively, the lack of immune genes upregulation in presence of Vd-CHIsal in the salivary blend could indicate that this gene may act directly as an immune response suppressor through an unrecognized mechanism which is worth of further studies.

The putative chitinolytic activity of Vd-CHIsal may be active against the chitinous cell wall of opportunistic saprophytic fungi, which are frequently vectored by the mites, such as, for example, *Aspergillus flavus*, the agent of stonebrood disease [51,52], or against the bacteria found associated with the feeding hole [17]. Unchecked fungal growth may cause blockage of the feeding site or a localized immune response in the host. Either scenario would reduce feeding success of mite and might explain the higher mortality we observed in RNAi-treated mites unable to produce a normal complement of Vd-CHIsal in the saliva. Fungi are not the only possible targets for Vd-CHIsal, because chitinases may also be active, directly or indirectly, towards bacteria. Although only few studies reported a direct antibacterial effect of chitinases [53], it is reasonable to speculate that Vd-CHIsal could also interfere with bacterial proliferation at the feeding site through production of antimicrobial chito-oligosaccharides derived from host’s chitin degradation [54] and by exerting anti-biofilm activity, as observed for other chitinases [55].

Collectively, these results clearly indicate that Vd-CHIsal is a major component of *V*. *destructor* saliva that plays an important role in the honey bee-mite interaction, and could be an amenable target for RNAi-based strategies of *Varroa* control. Moreover, we predict that the functional characterization of *Varroa* sialome will be a valuable approach towards the unraveling of the complex interactions at the parasite-host interface that modulate the virulence strategy of the mite and of vectored pathogens. This background knowledge will pave the way towards the promising opportunity to develop new bioinspired solutions for mite control, based on the disruption of the delicate mechanisms that *Varroa* uses for host colonization and its nutritional exploitation.

## Materials and methods

### Secretome prediction of *Varroa destructor*

Transcript sequences were downloaded from the *Varroa* genome database (Vdes_3.0, October 2017) available at NCBI (https://www.ncbi.nlm. nih.gov/genome/?term = varroa%20destructor) and in-house annotated following the Trinotate pipeline. Briefly, the transcript sequences were translated to amino-acid sequences using Transdecoder and blasted against the Uniprot/Swiss-Prot database, in order to obtain the Gene Ontology (GO) terms representing the whole predicted proteome of *V*. *destructor*.

In order to predict the secretome, the amino acid sequences were filtered for the presence of a signal peptide and the absence of transmembrane domains using SignalP v. 4.0 [56] and TMHMM v. 2.0 [57], respectively. To identify potential orthologs of regulatory factors present in arthropod secretions, the resulting subset of putatively secreted proteins was blasted against the following databases: venom proteins of Hymenoptera species available at NCBI (using keyword ‘venom AND Hymenoptera [organism]’); “saliva-related” tick’s and mite’s proteins available at NCBI (using keyword ‘saliva AND Acarina [organism]’).

For BLAST searches we used an E value cut-off of 10^−5^. Venn diagram of annotated proteins was plotted using the online tool Venny v. 2.1. GO terms from whole predicted proteome and secretome were compared and plotted using WEGO v. 2.0 [58].

### Biological material

*Varroa* mites and honey bees used in this study were collected from brood combs of *A*. *mellifera* colonies maintained at the experimental apiaries of the Department of Agricultural Sciences (University of Napoli Federico II), based in Portici (Napoli, Italy), and of the University of Aberdeen, based in Newburgh (Aberdeenshire, UK). Brood frames were collected between June and September and stored at 32 ± 1°C, 40 ± 2% relative humidity, under dark conditions, up to 24–48 h. Sealed brood cells were uncapped and worker pupae (white-eye stage) and adult females of *V*. *destructor* collected.

### Salivary glands dissection

*Varroa* adult females were collected and stuck ventrally onto a microscopy slide using double-sided sticky tape. Each mite was incised in the posterior and lateral region and submerged in 30 μL of PBS 1x. Using fine tweezers and dissection needles, the mite’s carapace was gently lifted-up and the salivary glands were isolated from other tissues, under a stereoscope (Discovery v8, Carl Zeiss Meditec AG, Jena, Germany). Pools of 5–10 mites were processed as above and the dissected tissues were stored at– 80°C in 200 uL of TRIzol Reagent (Thermo Fisher Scientific, *Waltham*, *MA*, *USA*).

### RNA isolation and Quantitative PCR (qRT-PCR) analysis

RNA isolation was performed using TRIzol Reagent (Thermo Fisher Scientific), according to the manufacturer’s instructions. RNA concentration and purity were assessed by measuring absorbance with Varioskan Flash (Thermo Fisher Scientific). Differential relative expression of genes putatively expressed in salivary glands was evaluated by one-step qRT-PCR, using the Power SYBR Green RNA-to-Ct 1-Step Kit (Applied Biosystems, Carlsbad, CA, USA), according to the manufacturer’s instructions. Each sample was prepared in 20 μL total volume containing 10 μL of qRT-PCR 2X reaction mix, forward and reverse primers at 100 nM final concentration, 0.16 μL of 125X RT enzyme mix, DEPC treated water and 50 ng of DNase-treated total RNA. For the experimental run the following thermal profile was used: 48°C for 30 min (RT); 95°C for 10 min; 40 cycles at 95°C for 15 s and 1 min at 58°C; a last cycle consisting of 15 s at 95°C, 60s at 58°C and 15 s at 95°C was added for carrying out a dissociation curve. Each sample was analyzed in triplicate on a Step One Real Time PCR System (Applied Biosystems). The 18S gene of *V*. *destructor* (Accession Number: XM_022831401.1) was used as endogenous control for RNA loading [59]. Relative gene expression data were analyzed using the ΔΔCt method [60]. For validation of the ΔΔCt method the difference between the Ct values of the target and the 18S transcripts [ΔCt = Ct(target)-Ct (18S)] was plotted versus the log of five-fold serial dilutions (100, 20, 4, 0.8 and 0.16 ng) of the purified RNA samples. The plot of log total RNA input versus ΔCt displayed a slope less than 0.1, indicating that the efficiencies of the two amplicons were approximately equal. The results are presented as mean fold changes of three independent biological replicates. Primers for *Vd-CHIsal* (S6 Table) were designed outside the dsRNA fragment used in silencing experiments.

Differential relative expression of selected immune genes of *A*. *mellifera* was measured by one-step qRT-PCR, as described above. qRT-PCR reactions were carried out using 50 ng of DNAse-treated RNA as input. Two reference genes, *β-actin* and *rps5*, were used as endogenous controls for RNA loading according to previous studies [61,62]. Primers for amplifying three out of four immune genes were selected based on literature data, as shown in S7 Table. Primers for *IRP30* were designed using Primer Express version 1.0 software (Applied Biosystems). The method of analysis was validated as described above and the efficiencies of the amplicons were approximately equal.

The quantification of DWV genome copies in individual honey bee pupae was performed by relating the Ct values of unknown samples to an established standard curve, as described elsewhere [10].

### Digoxigenin labeled probe synthesis

Total RNA was isolated with TRIzol Reagent from a pool of 10 adult female mites and retro-transcribed with High Capacity cDNA RT Kit according to manufacturer’s instructions (Thermo Fisher Scientific). Specific primers were designed in order to amplify a 300 bp fragment of *Vd-CHIsal* (soCHI300F: TCAAGGCAGATTCACCGAGTT; soCHI300R: ACCCATTGGTTTTGTTTATATGCA). The PCR reaction mix contained 25 μL of 2X DreamTaq Green PCR Master Mix (Thermo Fisher Scientific), the two primers at 500 nM final concentration, 2 μL of cDNA template and nuclease-free water to a total volume of 50 μL. The following cycling conditions were used for cDNA amplification: initial denaturation at 95°C for 3 minutes; 40 cycles of 95°C for 30 seconds, 53°C for 30 seconds and 72°C for 1 minute; final extension at 72°C for 10 minutes. The amplified product was inserted into the pCRII-TOPO vector, using the TA Cloning kit (Thermo Fisher Scientific). Following transformation of OneShot TOP10 chemically competent *E*. *coli* cells (Thermo Fisher Scientific), positive clones were selected by PCR reactions performed on plasmid DNA purified using the Pure Link HQ Mini Plasmid kit (Thermo Fisher Scientific). Plasmid DNA was linearized by restriction endonuclease digestion and used as template for *in vitro* synthesis of “run off” sense and antisense transcripts labeled with DIG-11-UTP (Roche Applied Science, Indianapolis, IN, USA). After treatment with DNAse I, labeled RNA molecules were precipitated with lithium chloride and resuspended in hybridization solution (see below). Labeling efficiency was evaluated by a spot test, where serial dilutions of the labeled RNA sample were applied to a positively charged membrane, along with known dilutions of a labeled control RNA serving as standard, and processed for immunological detection.

### *In situ* hybridization

*Varroa* adult females were incised in the posterior and lateral region, as described above, and immediately placed in fixative solution (4% paraformaldehyde, 0,1% TRiTON X-100 in PBS, pH 7.2) for overnight incubation at 4°C. Mites were rinsed 3 times in PBS and 2 times in nuclease-free water. Tissue dehydratation was carried out at room temperature through a series of increasing ethanol concentrations (30%, 50%, 70%, 80%, 90%, and 100%); dehydrated samples were placed in a solution of 50% ethanol: 50% xylol, subsequently replaced by a solution of 25% ethanol: 75% xylol and, finally, 100% xylol, three times, to remove completely the ethanol from the tissues. Paraplast chips were added gradually to the glass vials containing the samples that were finally placed in an oven at 60°C overnight. *Varroa* adults were oriented into embedding molds containing paraffin and were allowed to solidify for 2 h at room temperature. Paraffin blocks were stored at 4°C until use. Polymerised samples were removed from embedding molds and sectioned to 3–5 um on a Reichert Jung 2030 microtome. Tissue sections were placed on positively charged slides, dried at 37°C to promote optimal tissue adhesion to the slide and subsequently stored in dry boxes until use. To remove paraffin from the samples, the slides were inserted into slide racks and then placed into staining dishes containing 100% xylol and gently stirred twice for 15 min. Slides were removed from xylol and rinsed twice in 100% ethanol. Tissue sections were then rehydrated into ethanol series (100% to 30%), rinsed in distilled water three times, treated with Nonidet P-40 (1%) for 10 min and acetylated with 0.33% (vol/vol) acetic anhydride in 0.1 M triethanolamine-HCl (pH 8.00) for 15 min prior to hybridization. The sections were prehybridized for 1 hour at 65°C in prehybridization solution (50% formamide, 5x SSC, 40 μg/mL sonicated salmon sperm DNA) and incubated overnight at 60°C in hybridization buffer containing the DIG-11-UTP labeled probe to a concentration of 100 ng/mL. After hybridization, the sections were washed twice in 50% formamide, 2x SSC, 0.1% Tween-20, at 55°C for 1 hour. The hybridization signals were detected by an enzyme-linked immunoassay using alkaline phosphatase (AP)-labeled sheep anti-DIG antibody conjugate (Roche Applied Science) and p-nitroblue tetrazolium chloride (NBT)/ 5-bromo-4-chloro-3-indolyl phosphate (BCIP) substrate mixture. The antibody conjugate was added to the tissue sections and incubated in a humid chamber at 4°C overnight. The slides were rinsed three times with washing buffers. Colour development was performed by adding the chromogenic substrate to the tissue sections and incubating for 4 h at room temperature in the dark. The colour reaction was stopped by a 5 min wash in 0.1 M Tris-EDTA pH 8.0. The sections were dehydrated through successive incubation in ethanol (50%, 70%, 95%, and 100%) and xylol (twice for 15 min each) and mounted in Eukitt resin.

*In situ* hybridized slides were observed under a light microscope (Eclipse Ni-U, Nikon, Tokyo, Japan) and photographed with a digital camera (DS-Ri1 Nikon). Dark blue staining indicated where the DIG-labeled probe bound directly the mRNA of interest.

### Vd-CHIsal sequence analysis and phylogenetic tree reconstruction

The amino acid sequence of Vd-CHIsal was analysed using several online tools, such as Compute pl/Mw and Interproscan. BLASTp searches were performed using the amino acid sequence of Vd-CHIsal as a query against non-redundant Acarina (mites and ticks) and Hymenoptera NCBI databases, as well as Swiss-Prot database. Top BLAST hits were selected to obtain a set of Vd-CHIsal putative homologs from different taxonomic areas, which was enriched with other chitinase sequences derived from parasitoid-host studies. These sequences were chitinases from the following species: *Chelonus inanitus* (GenBank: CBM69270.1), *Chelonus* sp. near *curvimaculatus* (GenBank: AAA61639.1), *T*. *nigriceps* (GenBank: AAX69085.1) and *N*. *vitripennis* (GenBank: NP_001128139.2). Protein sequences were aligned and phylogeny reconstruction was carried out as reported elsewhere [31].

### dsRNA production and administration

Gene-specific primers flanked by T7 promoter sequences (F: TAATACGACTCACTATAGGGAGTAGGGCTTGCTTACGATG; R: TAATACGACTCACTATAGGGAGATATGCATAAGGTGTCTTGGA) were designed in order to amplify a 454 bp fragment of *Vd-CHIsal* (XM_022817406.1), which is not targeted by the qRT-PCR primers used to evaluate the level of *Vd-CHIsal* expression in salivary glands and in gene knock-down experiments (S6 Table). The amplified region was used as query in BLASTn searches, to assess the potential risk of dsRNA off-target effects towards *V*. *destructor* and *A*. *mellifera*. BLASTn analyses revealed that the most similar transcript to T7-flanked *Vd-CHIsal* sequences in *V*. *destructor* is another endochitinase (XM_022805355.1), but with a low coverage value (17% query cover, 82% identity, E value = 6e-04). In *A*. *mellifera* no significant similarity was found. cDNA from adult females of *V*. *destructor* was obtained as described above and used as template (2 μL) for PCR reactions containing the following components: 25 μL Phusion Flash High-Fidelity PCR Master Mix (Thermo Fisher Scientific), the two primers at 500 nM final concentration and nuclease-free water to a total volume of 50 μL. The cycle conditions were: 10 s at 98°C; 1s at 98°C, 5 s at 66°C and 15 s at 72°C for 5 cycles; 1s at 98°C and 15 s at 72°C for 30 cycles. Four samples were assembled to produce at least 1 μg of the amplicon, the minimum required for subsequent dsRNA synthesis. 5 μL of the amplified products were run on a 1% agarose gel to verify that the PCR reaction produced a single band of the expected size. The amplified products were then purified with PureLink PCR Purification Kit (Thermo Fisher Scientific), eluted in nuclease-free water and quantified measuring the absorbance with Varioskan Flash (Thermo Fisher Scientific). Following the manufacturer’s instructions (MEGAscript RNAi kit, Thermo Fisher Scientific), the transcription reactions were assembled using 1.2 μg of purified PCR product. After DNA and ssRNA digestion, the dsRNA was purified and eluted in 50 μL of a 0.9% NaCl solution. The dsRNA concentration was determined spectrophotometrically and the quality checked on a 1% agarose gel. A GFP dsRNA, exploited in control experiments, was similarly produced starting from the cloning vector pcDNA 3.1/CT-GFP TOPO (Thermo Fisher Scientific), which was used as template for a PCR reaction, performed as described elsewhere [63].

The dsRNA administration was performed by introducing 10 mites in a 1.5 mL tube containing 20 μL of 1 μg/μL dsRNA in saline solution (0.9% NaCl) or only saline solution for controls [64]. The tube was gently shaken, to drop the mites into the solution, and kept at room temperature for approximately 8 h. Every 30 minutes mites were checked to be sure that they were all submerged in the solution, where they usually remain stuck at the bottom of the tube. At the end of the immersion, mites were removed from the tubes using a fine brush and dried on filter paper.

### Artificial infestation

Groups of 10 mites were soaked and dried as described above. Then, each mite was individually introduced in a transparent gelatin capsule (6.5 mm) containing a single honey bee worker pupa. The capsules were perforated using a syringe needle to allow gas exchange and to prevent moisture accumulation, and fixed to the bottom of a petri dish using double-sided sticky tape, so that pupae were laying on their dorsum [65]. Artificial infestation was performed at 32°± 1°C, 80 ± 2% relative humidity and in the dark to simulate the hive environment.

### Time course analysis of *Vd-CHIsal* knockdown

In order to evaluate the level of *Vd-CHIsal* silencing over time, pools of 2–3 mites were sampled from each experimental group at 12, 24, 48 and 72 h after dsRNA immersion and stored at -80°C. Total RNA was isolated, DNAse-treated, quantified and used for qRT-PCR as described above.

### Survival of *V*. *destructor* after *Vd-CHIsal* gene knockdown

In order to measure the effects of the gene silencing on *Varroa* survival, each treated mite was introduced in a gel capsule containing 1 worker pupa (white eyes), as described above. *Vd-CHIsal* knocked-down and control mites were fed on an individual pupa for the entire duration of the bioassay or, in a separate set of trials, every 24 h the honey bee pupa was replaced. The different mite samples were placed in the same incubator, inside separate petri dishes, which were kept at the bottom of a humid chamber (32 ± 1°C, 80 ± 2% relative humidity). Mite survival was monitored every 24 h, by checking the motility of individuals. Each trial involved groups of 10–20 mites per treatment and was repeated three times. Survival rates and Kaplan-Meier estimators were determined using the software GraphPad Prism 7.

### RNA seq analysis of honey bee pupae transcriptome as affected by mites treated with *Vd-CHIsal* dsRNA

The white-eyed worker pupae exposed to mite feeding for 24 hours in the survival experiment described above, along with synchronous non-infested control pupae, were collected and stored at -80°C. Three biological replicates per treatment (non-infested control pupae; pupae infested with mites soaked in 0.9% NaCl, or in *Vd-CHIsal* dsRNA) were processed for RNAseq analysis. All the bees used in this experiment were obtained from the same hive. DWV genome copies were quantified by RT-qPCR as described elsewhere [10], and indicated an approximately equal infection level between treatments (~10^7^). Approximately 200 ng of total RNA was used for cDNA sequencing libraries construction with TruSeq stranded kit (Illumina, San Diego, CA, USA) and the 150 bp paired-end sequencing run was performed on the Illumina HiSeq1500 platform. The reads were cleaned from adapters, trimmed and mapped to the genome of *A*. *mellifera* (Amel_4.5), using the A.I.R. software (Sequentia Biotech, Barcelona, Spain). Differentially expressed genes were detected using the DESeq2 package [66], through the A.I.R software (Sequentia Biotech). FPKM (fragments per kilobase per million mapped fragments) values were used to perform a principal component analysis (PCA) (S3 Fig) and to check biological replicates of experimental and control samples. Fold-changes were reported as log (base 2) of normalized read count abundance for the Vd-CHIsal depleted samples divided by the read count abundance of the whole-saliva infested samples.

The raw sequences are available at the NCBI Sequence Read Archive (accession no. PRJNA628981).

### Statistical analysis

To assess differential expression in salivary glands ΔCts were compared using Student’s t-test. To validate *Vd-CHIsal* knockdown in *Varroa* mites and to evaluate differential expression of selected genes in honey bees, we used one-way analysis of variance (ANOVA) followed by Tukey’s test. In all cases statistical significance was set at P<0.05. The assumption of normal distribution of data was tested and met via Shapiro-Wilk test. Each dataset was checked for homoscedasticity using Levene’s test. For DWV titers, when ANOVA assumptions were not fulfilled, nonparametric Kruskal–Wallis ANOVA was used. All statistical analyses were performed using SPSS (IBM, Armonk, NY, USA). Data of *V*. *destructor* survival tests were analyzed using the software GraphPad Prism 7. The log rank (Mantel-Cox test) was used to compare the survival distributions of the observed groups and statistical significance was set at 0.05, when only one comparison was performed, or at 0.016, due to Bonferroni correction, for multiple comparisons.

## Supporting information

S1 Fig Amino acid sequence alignment of chitinases from different arthropod species. Vd-CHIsal (highlighted with a red line) is aligned with a putative paralog (47.84% identity, 77% query cover) of the same organism (*Varroa destructor*, XP_022661090.1) and with chitinases from the following species: the mites *Tropilaelaps mercedesae* (OQR72877.1) and *Galendromus occidentalis* (XP_003747412.1); the parasitic wasps *Chelonus inanitus* (CBM69270.1) and *Toxoneuron nigriceps* (AAX69085.1). The conserved motif of the glycoside hydrolase family 18 (DXXDXDXE) containing the E147 active site is boxed in red. Locations of the catalytic and chitin-binding domains are indicated by blue and green lines, respectively. Red and green arrows indicate the beginning of the predicted signal peptide and mature protein sequences of Vd-CHIsal, respectively. Amino acid colors follow the Clustal X color scheme: hydrophobic residues are in blue, positively charged residues are in red, negatively charged residues are in magenta, polar residues are in green, cysteines are in pink, glycines are in orange, prolines are in yellow, aromatic residues are in cyan and non-conserved residues are in white.
(PDF)

S2 Fig DWV titers in honey bee pupae used for transcriptional analyses and in survival assays of mites. Log scale values of DWV genome copies registered in individual honey bee pupae, for each experimental group considered (NP: non-parasitized controls; WS: pupae infested with mites soaked in saline solution; KD: pupae infested with mites soaked in *Vd-CHIsal* dsRNA solution; GFP: pupae infested with mites soaked in GFP dsRNA solution), are reported. The line indicates the mean value, which did not differ among the different experimental conditions.
(PDF)

S3 Fig Principal component analysis of normalized RNA-seq data. FPKM (fragments per kilobase per million mapped fragments) values were used to perform a principal component analysis. Colored dots represent individual biological replicates. Honey bee pupae infested by mites with whole salivary repertoire are indicated by blue circles, while honey bee pupae infested by mites with reduced levels of Vd-CHIsal are indicated by red squares.
(PDF)

S1 Table Putative homologs of ticks’ salivary proteins in *V*. *destructor* candidate host-regulation factors. In order to define a first set of putative salivary effectors to be studied from a functional point of view, we mined literature relative to the well-studied salivary blends of ticks and selected candidate host regulation factors. We then used BLAST to infer putative homologies in *V*. *destructor* secreted proteins having a match in both saliva of Acarina and venom of Hymenoptera. We focused on top three hits, with the lowest E-values: α-Macroglobulin, Aspartic Protease and Chitinase. These potential host regulation factors were also found in an in-house proteomic atlas of *V*. *destructor* saliva, available at the University of Aberdeen.
(PDF)

S2 Table ANOVA and Tukey’s post hoc test comparisons of ΔCt (Ct *Vd-CHIsal*–Ct *18S*) values. (PDF)

S3 Table Genes downregulated in honey bee pupae upon infestation with *Varroa* mites injecting Vd-CHIsal deficient saliva. To investigate the possible effects of Vd-CHIsal on honey bees, we studied the host transcriptional response upon infestation with *Varroa* mites producing Vd-CHIsal deficient saliva (KD), compared to control mites, delivering saliva with the whole repertoire of virulence factors (WS). Non-parasitized control pupae (NP) were used as reference sample. Fold-changes (FC) were reported as log (base 2) of normalized read count abundance for the Vd-CHIsal depleted samples divided by the read count abundance of the whole-saliva infested samples. DESeq2 adjusted *P* was < 0.05 and FDR was set at 5%.
(PDF)

S4 Table Genes upregulated in honey bee pupae upon infestation with *Varroa* mites injecting Vd-CHIsal deficient saliva. To investigate the possible effects of Vd-CHIsal on honey bees, we studied the host transcriptional response upon infestation with *Varroa* mites producing Vd-CHIsal deficient saliva (KD), compared to control mites, delivering saliva with the whole repertoire of virulence factors (WS). Non-parasitized control pupae (NP) were used as reference sample. Fold-changes (FC) were reported as log (base 2) of normalized read count abundance for the Vd-CHIsal depleted samples divided by the read count abundance of the whole-saliva infested samples. DESeq2 adjusted *P* was < 0.05 and FDR was set at 5%.
(PDF)

S5 Table ANOVA and Tukey’s post hoc test comparisons of ΔCt values. (PDF)

S6 Table Primers used for qRT-PCR survey of candidate salivary effectors. (PDF)

S7 Table qRT-PCR primers used for studying the transcriptional profile of honeybee immune genes. (PDF)

S1 File Table resulting from Trinotate in-house annotation of the putatively secreted components of *Varroa destructor* predicted proteome. The annotation was performed following the protocol described in Materials and Methods section. Content of columns is hereafter described. A: GenBank accession number of the original transcript of *V*. *destructor*; B: GenBank accession number of the matching protein in *V*. *destructor*; C: ID name assigned by Transdecoder software; D: coordinates of the translation; E: top BLASTp hits in Swiss-Prot db; F: top BLASTp hits in the database of Hymenoptera venom; G: top BLASTp hits in the database of Acarina saliva; H: Pfam hits; I,: SignalP results indicating the presence of the signal peptide; J: TMHMM results indicating the presence of transmembrane domains; K: eggnog db hits; L: Kegg db hits; M: gene ontology retrieved from BLASTp; N: gene ontology retrieved from Pfam.
(XLSX)
