# Supplementary material for: A salivary chitinase of Varroa destructor influences host immunity and mite’s survival
Source: PLoS Pathog. 2020 Dec 4;16(12):e1009075. doi: 10.1371/journal.ppat.1009075 (PMC7744053; doi:10.1371/journal.ppat.1009075)
Supplement: S1 Fig — Vd-CHIsal (highlighted with a red line) is aligned with a putative paralog (47.84% identity, 77% query cover) of the same organism (Varroa destructor, XP_022661090.1) and with chitinases from the following species: the mites Tropilaelaps mercedesae (OQR72877.1) and Galendromus occidentalis (XP_003747412.1); the parasitic wasps Chelonus inanitus (CBM69270.1) and Toxoneuron nigriceps (AAX69085.1). The conserved motif of the glycoside hydrolase family 18 (DXXDXDXE) containing the E147 active site is boxed in red. Locations of the catalytic and chitin-binding domains are indicated by blue and green lines, respectively. Red and green arrows indicate the beginning of the predicted signal peptide and mature protein sequences of Vd-CHIsal, respectively. Amino acid colors follow the Clustal X color scheme: hydrophobic residues are in blue, positively charged residues are in red, negatively charged residues are in magenta, polar residues are in green, cysteines are in pink, glycines are in orange, prolines are in yellow, aromatic residues are in cyan and non-conserved residues are in white. (PDF) [file ppat.1009075.s001.pdf]

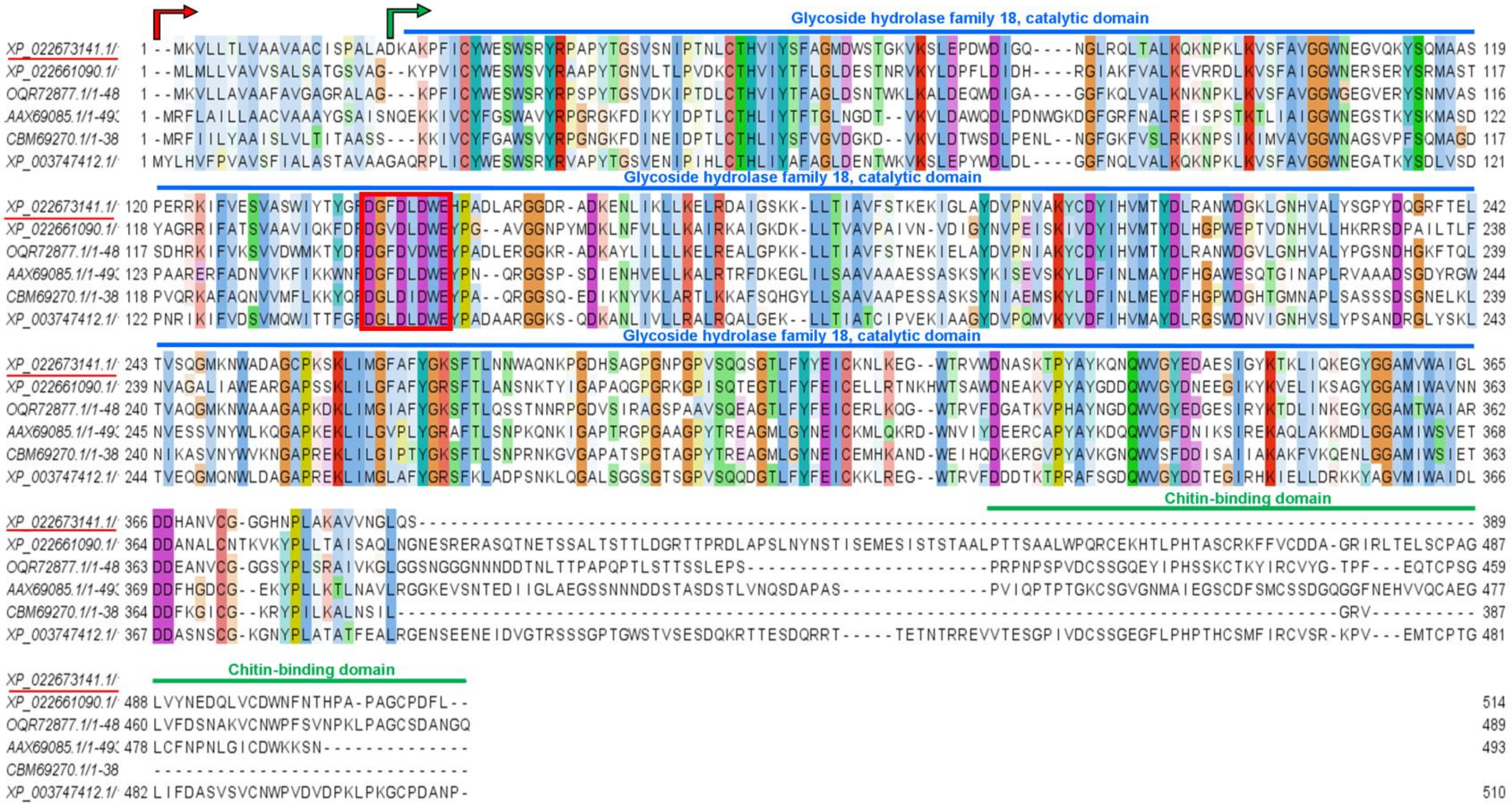

S1 Fig. Amino acid sequence alignment of chitinases from different arthropod species. Vd-CHIsal (highlighted with a red line) is aligned with a putative paralog (47.84% identity, 77% query cover) of the same organism (*Varroa destructor*, XP_022661090.1) and sequences of chitinases from the following species: the mites *Tropilaelaps mercedesae* (OQR72877.1) and *Galendromus occidentalis*

(XP_003747412.1); the parasitic wasps *Chelonus inanitus* (CBM69270.1) and *Toxoneuron nigriceps* (AAX69085.1). The conserved motif DXXDXDXE with the active site of GH18 (Glu147) is boxed in red. Locations of the glycoside hydrolase family 18 catalytic domain and chitin-binding domains are indicated by blue and green lines, respectively. Red and green arrows indicate the beginning of the predicted signal peptide and mature protein sequences of Vd-CHI, respectively. Amino acids colors follow the Clustal X color scheme: hydrophobic are blue, positively charged are red, negatively charged are magenta, polar are green, cysteines are pink, glycines are orange, prolines are yellow, aromatic are cyan and non-conserved are white.
