# Supplementary material for: A salivary chitinase of Varroa destructor influences host immunity and mite’s survival
Source: PLoS Pathog. 2020 Dec 4;16(12):e1009075. doi: 10.1371/journal.ppat.1009075 (PMC7744053; doi:10.1371/journal.ppat.1009075)
Supplement: S2 Fig — Log scale values of DWV genome copies registered in individual honey bee pupae, for each experimental group considered (NP: non-parasitized controls; WS: pupae infested with mites soaked in saline solution; KD: pupae infested with mites soaked in Vd-CHIsal dsRNA solution; GFP: pupae infested with mites soaked in GFP dsRNA solution), are reported. The line indicates the mean value, which did not differ among the different experimental conditions. (PDF) [file ppat.1009075.s002.pdf]

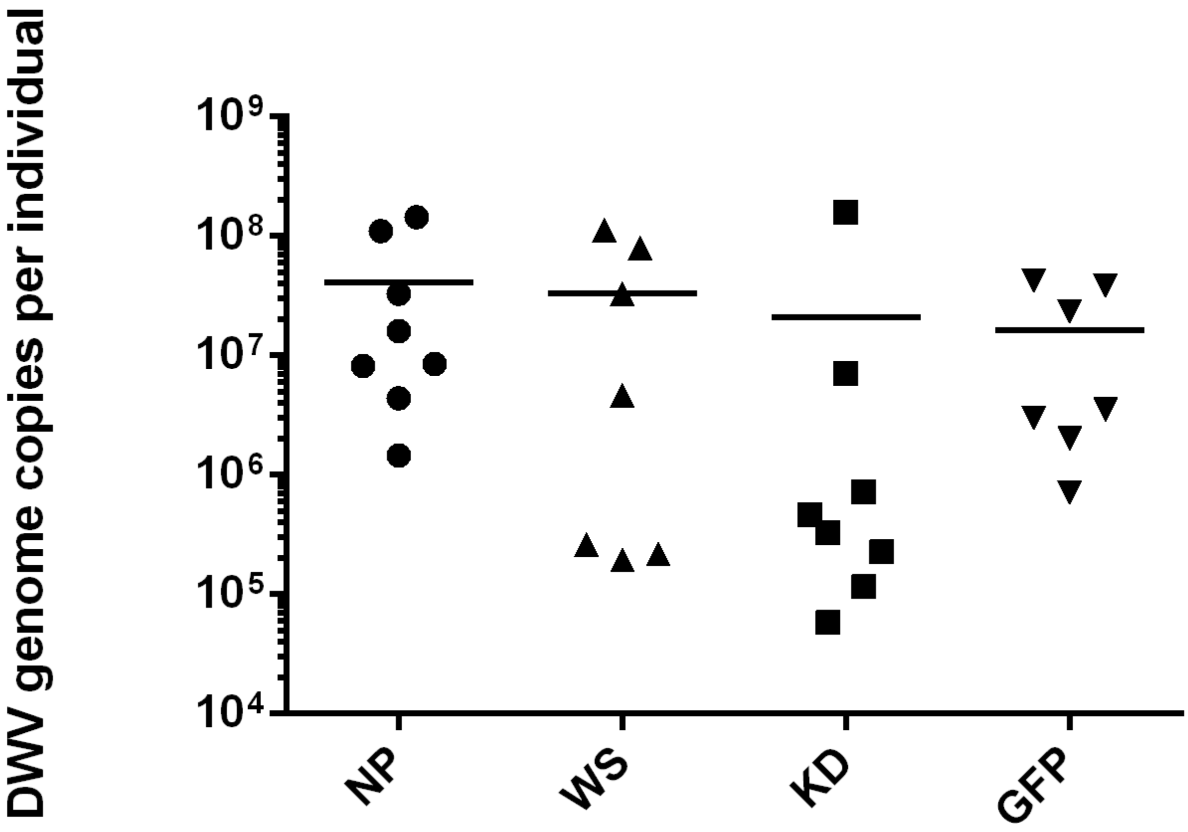

S2 Fig. DWV titers in honey bee pupae used for transcriptional analyses and in survival assays of mites. Log scale values of DWV genome copies registered in individual honey bee pupae, for each experimental group considered (NP: non-parasitized controls; WS: pupae infested with mites soaked in saline solution; KD: pupae infested with mites soaked in Vd-CHIsal dsRNA solution; GFP: pupae infested with mites soaked in GFP dsRNA solution), are reported. The line indicates the mean value, which did not differ among the different experimental conditions.
