# Supplementary material for: A salivary chitinase of Varroa destructor influences host immunity and mite’s survival
Source: PLoS Pathog. 2020 Dec 4;16(12):e1009075. doi: 10.1371/journal.ppat.1009075 (PMC7744053; doi:10.1371/journal.ppat.1009075)
Supplement: S3 Fig — FPKM (fragments per kilobase per million mapped fragments) values were used to perform a principal component analysis. Colored dots represent individual biological replicates. Honey bee pupae infested by mites with whole salivary repertoire are indicated by blue circles, while honey bee pupae infested by mites with reduced levels of Vd-CHIsal are indicated by red squares. (PDF) [file ppat.1009075.s003.pdf]

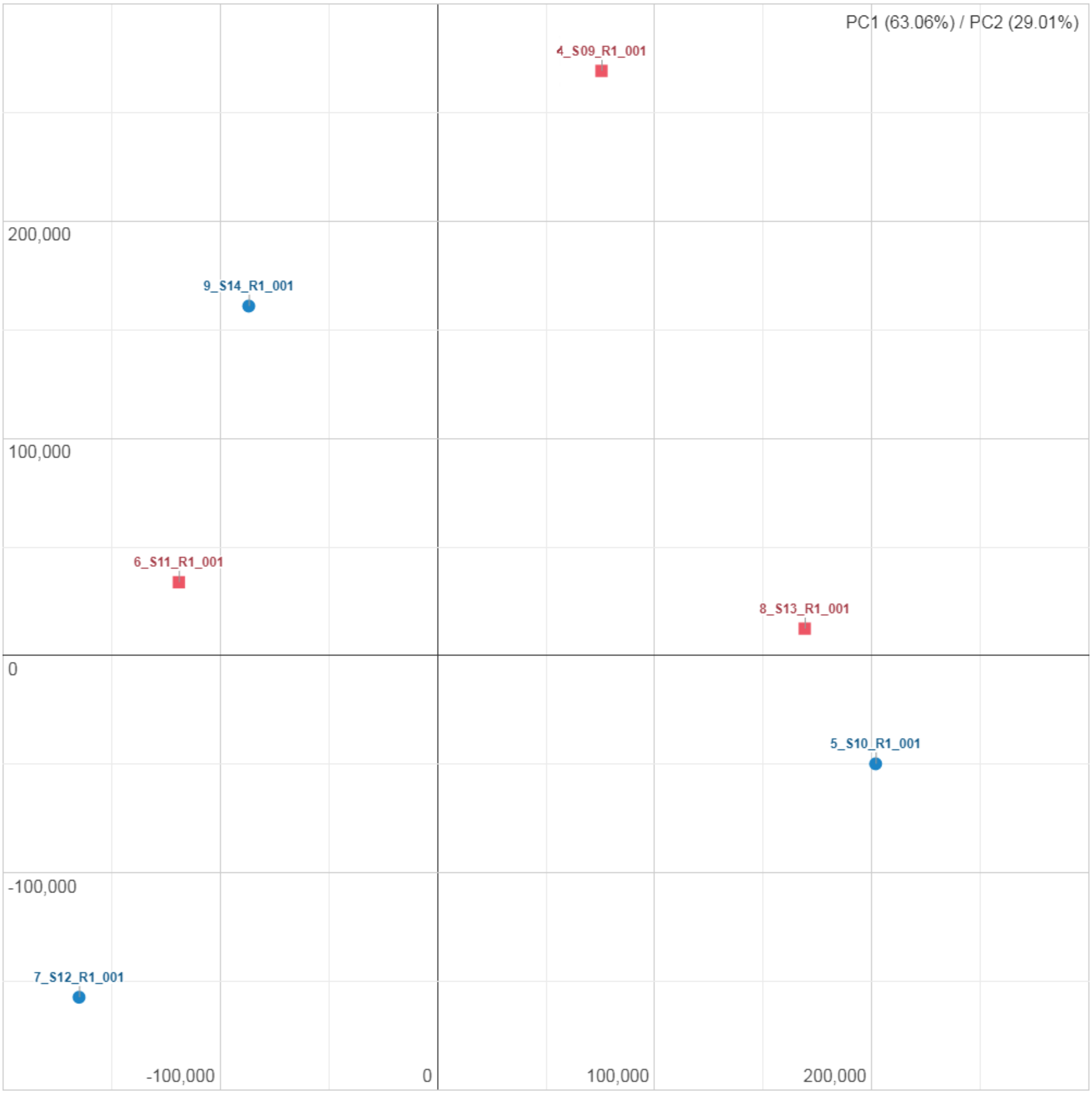

S3 Fig. Principal component analysis of normalized RNA-seq data. FPKM (fragments per kilobase per million mapped fragments) values were used to perform a principal component analysis. Colored dots represent individual biological replicates. Honey bee pupae infested by mites with whole salivary repertoire are indicated by blue circles, while honey bee pupae infested by mites with reduced levels of Vd-CHIsal are indicated by red squares.
