# Supplementary material for: A salivary chitinase of Varroa destructor influences host immunity and mite’s survival
Source: PLoS Pathog. 2020 Dec 4;16(12):e1009075. doi: 10.1371/journal.ppat.1009075 (PMC7744053; doi:10.1371/journal.ppat.1009075)
Supplement: S1 Table — In order to define a first set of putative salivary effectors to be studied from a functional point of view, we mined literature relative to the well-studied salivary blends of ticks and selected candidate host regulation factors. We then used BLAST to infer putative homologies in V. destructor secreted proteins having a match in both saliva of Acarina and venom of Hymenoptera. We focused on top three hits, with the lowest E-values: α-Macroglobulin, Aspartic Protease and Chitinase. These potential host regulation factors were also found in an in-house proteomic atlas of V. destructor saliva, available at the University of Aberdeen. (PDF) [file ppat.1009075.s004.pdf]

S1 Table. Putative homologs of ticks' salivary proteins in *V. destructor* candidate host-regulation factors

Protein	Salivary protein of tick	Reference	*V. destructor* putative homolog	E-value	Identity (%)
α-Macroglobulin	ACJ26770	[1]	XM_022809118	0.0	34.434
Aspartic protease	AAG00993	[1]	XM_022800952	7.63e-86	40.00
Chitinase	AIR95100	[2]	XM_022817406	4.52e-109	42.547
BPTI/Kunitz-type serine protease inhibitor	JAU02547	[3]	XM_022804925	1.97e-13	31.818
Metalloprotease	CAO00625	[4]	XM_022809866	4.64e-58	33.93
Serpin	AID54718	[5]	XM_022805957	4.10e-43	30.10
Kunitz-type protein	CAB55816	[6]	XM_022791924	1.13e-13	48.15
Multiple coagulation factor deficiency protein	BAI99729	[7]	XM_022816950	3.75e-29	36.55

In order to define a first set of putative salivary effectors to be studied from a functional point of view, we mined literature relative to the well-studied salivary blends of ticks and selected candidate host regulation factors. We then used BLAST to infer putative homologies in *V. destructor* secreted proteins having a match in both saliva of Acarina and venom of Hymenoptera. We focused on top three hits, with the lowest E-values: α-Macroglobulin, Aspartic Protease and Chitinase. These potential host regulation factors were also found in an in-house proteomic atlas of *V. destructor* saliva, available at the University of Aberdeen.

References

1. Tirloni L, Reck J, Terra RMS, Martins JR, Mulenga A, Sherman NE, et al. Proteomic Analysis of Cattle Tick Rhipicephalus (Boophilus) microplus Saliva: A Comparison between Partially and Fully Engorged Females. PLoS One. 2014;9. doi:10.1371/journal.pone.0094831

2. Kim TK, Curran J, Mulenga A. Dual silencing of long and short Amblyomma americanum acidic chitinase forms weakens the tick cement cone stability. Journal of Experimental Biology. 2014; jeb.107979. doi:10.1242/jeb.107979

3. Esteves E, Maruyama SR, Kawahara R, Fujita A, Martins LA, Righi AA, et al. Analysis of the Salivary Gland Transcriptome of Unfed and Partially Fed Amblyomma sculptum Ticks and Descriptive Proteome of the Saliva. Front Cell Infect Microbiol. 2017;7. doi:10.3389/fcimb.2017.00476

4. Decrem Y, Beaufays J, Blasioli V, Lahaye K, Brossard M, Vanhamme L, et al. A family of putative metalloproteases in the salivary glands of the tick Ixodes ricinus. The FEBS Journal. 2008;275: 1485–1499. doi:10.1111/j.1742-4658.2008.06308.x

5. Ibelli AMG, Kim TK, Hill CC, Lewis LA, Bakshi M, Miller S, et al. A blood meal-induced Ixodes scapularis tick saliva serpin inhibits trypsin and thrombin, and interferes with platelet aggregation and blood clotting. International Journal for Parasitology. 2014;44: 369–379. doi:10.1016/j.ijpara.2014.01.010

6. Decrem Y, Rath G, Blasioli V, Cauchie P, Robert S, Beaufays J, et al. Ir-CPI, a coagulation contact phase inhibitor from the tick Ixodes ricinus, inhibits thrombus formation without impairing hemostasis. J Exp Med. 2009;206: 2381–2395. doi:10.1084/jem.20091007

7. Anisuzzaman null, Islam MK, Miyoshi T, Alim MA, Hatta T, Yamaji K, et al. Longistatin, a novel EF-hand protein from the ixodid tick Haemaphysalis longicornis, is required for acquisition of host blood-meals. Int J Parasitol. 2010;40: 721–729. doi:10.1016/j.ijpara.2009.11.004
