# Supplementary material for: A salivary chitinase of Varroa destructor influences host immunity and mite’s survival
Source: PLoS Pathog. 2020 Dec 4;16(12):e1009075. doi: 10.1371/journal.ppat.1009075 (PMC7744053; doi:10.1371/journal.ppat.1009075)
Supplement: S2 Table — (PDF) [file ppat.1009075.s005.pdf]

S2 Table. ANOVA results and Tukey's post hoc test significativity of ΔCt (Ct *Vd-CHIsal* – Ct *18S*) comparison between groups

Gene name	Df[a]	F	Significativity	Post hoc test comparison	Tukey's test significativity[b]
12 h	2,18	0.145	P=0.866	0.9% NaCl - GFP dsRNA	-
				0.9% NaCl - Vd-CHIsal dsRNA	-
				GFP dsRNA - Vd-CHIsal dsRNA	-
24 h	2,18	3.231	P=0.063	0.9% NaCl - GFP dsRNA	-
				0.9% NaCl - Vd-CHIsal dsRNA	-
				GFP dsRNA - Vd-CHIsal dsRNA	-
48 h	2,18	23.01	P<0.001	0.9% NaCl - GFP dsRNA	-
				0.9% NaCl - Vd-CHIsal dsRNA	P<0.001
				GFP dsRNA - Vd-CHIsal dsRNA	P<0.001
72 h	2,18	15.32	P<0.001	0.9% NaCl - GFP dsRNA	-
				0.9% NaCl - Vd-CHIsal dsRNA	P<0.001
				GFP dsRNA - Vd-CHIsal dsRNA	P<0.001

[a] Degrees of freedom. The first value indicates degrees of freedom between the groups, while the second value, after the comma, indicates degrees of freedom within the groups.

[b] Non-significant post-hoc tests are indicated by "-".
