# Supplementary material for: A salivary chitinase of Varroa destructor influences host immunity and mite’s survival
Source: PLoS Pathog. 2020 Dec 4;16(12):e1009075. doi: 10.1371/journal.ppat.1009075 (PMC7744053; doi:10.1371/journal.ppat.1009075)
Supplement: S3 Table — To investigate the possible effects of Vd-CHIsal on honey bees, we studied the host transcriptional response upon infestation with Varroa mites producing Vd-CHIsal deficient saliva (KD), compared to control mites, delivering saliva with the whole repertoire of virulence factors (WS). Non-parasitized control pupae (NP) were used as reference sample. Fold-changes (FC) were reported as log (base 2) of normalized read count abundance for the Vd-CHIsal depleted samples divided by the read count abundance of the whole-saliva infested samples. DESeq2 adjusted P was < 0.05 and FDR was set at 5%. (PDF) [file ppat.1009075.s006.pdf]

S3 Table. Genes downregulated in honey bee pupae upon infestation with *Varroa* mites injecting Vd-CHIsal deficient saliva

Gene ID	FDR	P-value	logFC (WS)	logFC (NP)	Mean FPKM KD	Mean FPKM WS	Description
LOC409455	1.64e-09	3.63e-13	1.37		0.82	5.44	Doublesex- and mab-3-related transcription factor A2
LOC725470	2.34e-07	1.03e-10	1.16		1.87	6.45	Sialin; MFS-type transporter SLC17A5
LOC413112	0.0005	3.82e-07	0.93		0.44	3.52	Pyruvate kinase
LOC107964741	0.004	3.96e-06	0.85	1.58	2.99	15.25	ncRNA
LOC107964962	0.003	3.04e-06	0.80		0.47	6.93	MFS-type transporter SLC18B1
LOC724933	0.008	9.78e-06	0.62		38.79	63.42	Xanthine dehydrogenase

To investigate the possible effects of *Vd-CHIsal* on honey bees, we studied the host transcriptional response upon infestation with *Varroa* mites producing Vd-CHIsal deficient saliva (KD), compared to control mites, delivering saliva with the whole repertoire of virulence factors (WS). Non-parasitized control pupae (NP) were used as reference sample. Fold-changes (FC) were reported as log (base 2) of normalized read count abundance for the Vd-CHIsal depleted samples divided by the read count abundance of the whole-saliva infested samples. DESeq2 adjusted P was < 0.05 and FDR was set at 5%.
