# Supplementary material for: A salivary chitinase of Varroa destructor influences host immunity and mite’s survival
Source: PLoS Pathog. 2020 Dec 4;16(12):e1009075. doi: 10.1371/journal.ppat.1009075 (PMC7744053; doi:10.1371/journal.ppat.1009075)
Supplement: S4 Table — To investigate the possible effects of Vd-CHIsal on honey bees, we studied the host transcriptional response upon infestation with Varroa mites producing Vd-CHIsal deficient saliva (KD), compared to control mites, delivering saliva with the whole repertoire of virulence factors (WS). Non-parasitized control pupae (NP) were used as reference sample. Fold-changes (FC) were reported as log (base 2) of normalized read count abundance for the Vd-CHIsal depleted samples divided by the read count abundance of the whole-saliva infested samples. DESeq2 adjusted P was < 0.05 and FDR was set at 5%. (PDF) [file ppat.1009075.s007.pdf]

S4 Table. Genes upregulated in honey bee pupae upon infestation with *Varroa* mites injecting Vd-CHIsal deficient saliva

Gene ID	FDR	P-value	logFC (WS)	logFC (NP)	Mean FPKM KD	Mean FPKM WS	Description
LOC551369	1.99e-23	2.21e-27	1.49		274.70	78.22	Actin-like
LOC406142	2.34e-07	8.91e-11	1.10		1200.39	53.13	Hymenoptaecin
LOC552584	5.58e-05	3.08e-08	0.95		2.30	0.18	Topoisomerase 3
LOC406140	0.004	5.24e-06	0.76	-1.58	7.14	0.87	Apidaecins type 14
LOC100578816	0.03	4.35e-05	0.74		74.59	17.93	Unknown
LOC408807	0.001	9.85e-07	0.73		36.62	1.56	LRR (IRP30)
LOC406144	0.02	3.47e-05	0.69		283.98	44.38	Abaecin

To investigate the possible effects of *Vd-CHIsal* on honey bees, we studied the host transcriptional response upon infestation with *Varroa* mites producing Vd-CHIsal deficient saliva (KD), compared to control mites, delivering saliva with the whole repertoire of virulence factors (WS). Non-parasitized control pupae (NP) were used as reference sample. Fold-changes (FC) were reported as log (base 2) of normalized read count abundance for the Vd-CHIsal depleted samples divided by the read count abundance of the whole-saliva infested samples. DESeq2 adjusted P was < 0.05 and FDR was set at 5%.
