# Supplementary material for: A salivary chitinase of Varroa destructor influences host immunity and mite’s survival
Source: PLoS Pathog. 2020 Dec 4;16(12):e1009075. doi: 10.1371/journal.ppat.1009075 (PMC7744053; doi:10.1371/journal.ppat.1009075)
Supplement: S5 Table — (PDF) [file ppat.1009075.s008.pdf]

S5 Table. ANOVA results and Tukey's post hoc test significativity of ΔCt comparison between groups

Gene name	Df[a]	F	Significativity	Post hoc test comparison	Tukey's test significativity[b]
Abaecin	2,20	1.857	*P*=0.182	NP-WS	-
				NP-KD	-
				WS-KD	-
Apidaecin	2,22	5.993	*P*=0.01	NP-WS	-
				NP-KD	-
				WS-KD	*P*=0.015
Hymenoptaecin	2,26	22.08	*P*<0.001	NP-WS	*P*=0.035
				NP-KD	*P*<0.001
				WS-KD	*P*=0.038
Immune responsive protein 30	2,20	5.294	*P*=0.014	NP-WS	-
				NP-KD	*P*=0.043
				WS-KD	*P=0.023*

[a] Degrees of freedom. The first value indicates degrees of freedom between the groups, while the second value, after the comma, indicates degrees of freedom within the groups.

[b] Non-significant post-hoc tests are indicated by "-".
