# Supplementary material for: A salivary chitinase of Varroa destructor influences host immunity and mite’s survival
Source: PLoS Pathog. 2020 Dec 4;16(12):e1009075. doi: 10.1371/journal.ppat.1009075 (PMC7744053; doi:10.1371/journal.ppat.1009075)
Supplement: S6 Table — (PDF) [file ppat.1009075.s009.pdf]

S6 Table. Primers used for qRT-PCR survey of candidate salivary effectors.

Transcript	Sequence
α-Macroglobulin	F: GAGCCCATTGCTGCTGAAAG
(XM_022809116.1)	R: TGTATATTCTCATCTCCATCCTCGAA
Aspartic Protease	F: GCCCGTTTCCCGTCTGTT
(XM_022800952.1)	R: CATCGCCGAGAATCCAAAGA
Chitinase	F: GCTACGGTGGAGCGATGGT
(XM_022817406.1)	R: TGACCTCCGCCACAGACAT
18S	F: AATGCCATCATTACCATCCT
(XM_022831401.1)	R: CAAAAACCAATCGGCAATCT
