# Supplementary material for: A salivary chitinase of Varroa destructor influences host immunity and mite’s survival
Source: PLoS Pathog. 2020 Dec 4;16(12):e1009075. doi: 10.1371/journal.ppat.1009075 (PMC7744053; doi:10.1371/journal.ppat.1009075)
Supplement: S7 Table — (PDF) [file ppat.1009075.s010.pdf]

S7 Table. qRT-PCR primers used for studying the expression profile of honeybee immune response genes

Transcript	Sequence	Gene description	Reference
Abaecin (NM_001011617.1)	F: CAGCATTCGCATACGTACCA<br>R: GACCAGGAAACGTTGGAAAC	Immune; antimicrobial peptide	[1]
Apidaecin (X72577.1)	F: TTTTGCCTTAGCAATTCTTGTTG<br>R: GTAGGTCGAGTAGGCGGATCT	Immune; antimicrobial peptide	[2]
Hymenoptaecin (NM_001011615.1)	F: CTCTTCTGTGCCGTTGCATA<br>R: GCGTCTCCTGTCATTCCATT	Immune; antimicrobial peptide	[1]
IRP30 (XM_397526.7)	F: GTAATCGGATCACGGAAATAGA<br>R: GTGAGCGATGTCCCAAAC	Immune; leucine-rich repeat protein	This study
β-Actin (NM_001185145.1)	F: TGCCAACACTGTCCTTTCTG<br>R: AGAATTGACCCACCAATCCA	House-keeping, cytoskeletal structural protein	[3]
RPS5 (XM_006570236.3)	F: AATTATTTGGTCGCTGGAATTG<br>R: TAACGTCCAGCAGAATGTGGTA	House-keeping, Ribosomal protein S5	[1]

References

1. Evans JD. Beepath: an ordered quantitative-PCR array for exploring honey bee immunity and disease. J Invertebr Pathol. 2006;93: 135–139. doi:10.1016/j.jip.2006.04.004

2. Simone M, Evans JD, Spivak M. Resin Collection and Social Immunity in Honey Bees. Evolution. 2009;63: 3016–3022. doi:10.1111/j.1558-5646.2009.00772.x

3. Lourenço AP, Mackert A, Cristino A dos S, Simões ZLP. Validation of reference genes for gene expression studies in the honey bee, Apis mellifera, by quantitative real-time RT-PCR. Apidologie. 2008;39: 372–385. doi:10.1051/apido:2008015
